# Incorporating correctional medicine exposure into pre-health education: a program description

**DOI:** 10.5116/ijme.6ac6.3840

**Published:** 2026-10-22

**Authors:** Sebastian Herrera Vargas, Sophia L. Jansen, Mirabella Romano, Emma Coatsworth, Hsien Chiang, Eric Handler, Jonathan Watson, Sharief Taraman

**Affiliations:** 1 Rady Children's Health Orange County, Sharon Disney Lund Medical Intelligence, Information, Investigation, and Innovation Institute, USA; 2 California Advancing and Innovating Medi-Cal, CalOptima Health, USA; 3 Independent Researcher, Santa Ana, USA; 4 Correctional Health Services, Orange County Healthcare Agency, USA

**Keywords:** Correctional medicine, health professions education, experiential learning, health equity, structural determinants of health

## Introduction

Recent discussions in the International Journal of Medical Education, including the scoping review by Swami and colleagues, highlight gaps in how structural determinants of health are taught in the United States.^1^ Correctional medicine is an essential yet persistently underrepresented domain within these discussions. Carceral settings function as de facto safety-net healthcare systems for millions of individuals annually and disproportionately serve patients with high burdens of chronic disease, mental illness, substance use disorders, and unmet preventive care needs.^2^ Despite this, formal education on healthcare delivery in correctional environments remains largely absent from pre-health curricula.^3^ As a result, many future clinicians enter practice with a limited understanding of how incarceration, legal status, and institutional constraints shape both patient outcomes and clinical decision-making.

Correctional healthcare is distinguished from traditional clinical environments by a combination of clinical complexity, structural constraint, and ethical questions that are rarely encountered simultaneously elsewhere. Individuals who are incarcerated frequently present with advanced or poorly controlled diseases. Recent data from the Bureau of Justice Statistics indicate that human immunodeficiency virus prevalence among incarcerated populations remains substantially higher than in the general population, reflecting structural inequities in preventive and primary care access.^4^ Care delivery is limited by security protocols, restricted patient movement, and coordination requirements between healthcare and corrections staff. Beyond managing multimorbid, high-acuity presentations, these clinicians must understand structural determinants of health, including mass incarcerations and racialized policing, that shape patient health before, during, and after incarceration.^5^

Experiential learning within correctional settings offers one mechanism to address this gap by contextualizing health disparities, challenging stigma-driven assumptions, and reframing correctional facilities as integral points of healthcare access.^6^ The Medical Intelligence, Information, Investigation, and Innovation (Mi4) Summer Internship sought to operationalize this approach by integrating guided exposure to correctional medicine into its pre-health curriculum for its Summer 2024 and 2026 cohorts. The experience involved supervised observation of correctional facility environments, structured discussion with practicing correctional physicians, and dialogues addressing challenges in clinical workflows.

A total of 61 interns participated in the correctional facility visit, of which twenty-five participated in 2026 and thirty-six participated in 2024. Voluntary post-visit surveys were completed by 86.9% (53/61) of participants with a 100% (25/25) completion rate for those who participated in 2026 and 77.8% (28/36) for those who participated in 2024. Themes and statistics were derived from survey responses describing what interns observed at the correctional facility and the group discussion held at the conclusion of the visit. No formal coding framework was applied. This project constituted an educational program evaluation and did not involve the collection of identifiable human subject data; thus, institutional review board approval was not required.

The survey incorporated both a structured rating scale and open-ended questions addressing prior exposure to correctional facilities, baseline familiarity with correctional healthcare, perceptions before the tour, and reflections afterward. Responses were analyzed descriptively and reviewed for recurring themes.

Out of the 53 respondents across both cohorts, 90.6% reported no previous educational or experiential exposure to correctional facilities (48/53). Baseline familiarity with correctional healthcare averaged 1.71 on a five-point scale (1.71/5), while the reported change in understanding following the tour averaged 4.38 on a five-point scale (4.38/5), with 79.2% of the respondents (42/53) indicating the tour challenged a prior assumption.

Our analysis found that respondents in both cohorts shared similar themes. Some preconceptions commonly anticipated were minimal healthcare resources, limited staffing, and highly restrictive environments. After the tour, respondents described the facility as more complex and structured than expected, coming to view these facilities as complex clinical environments that serve as essential access points for marginalized populations. Specific protocols regarding security and transportation logistics present constraints that could also represent opportunities for investment and innovation. Several respondents across both cohorts independently proposed similar technology-based solutions, most notably contraband safe continuous glucose monitoring and AI assisted electronic health record integration, underscoring a recurring perception that administrative and technological infrastructure represents a key barrier to care continuity.

These reflections should be interpreted as possible benefits rather than confirmed outcomes. They derive from two cohorts, totaling fifty-three survey responses, and self-reported reflections collected without a pre-visit assessment or comparison group. Thus, we cannot determine whether the reported lessons reflect measurable change in empathy or attitude. Prospective designs incorporating pre- and post-visit measurement, longitudinal follow-up, and a comparison group would be necessary to draw firm conclusions about long-term effects.

At the program level, the Mi4 Internship is designed to be scalable. Future cohorts aim to deepen structured reflection, strengthen longitudinal engagement with correctional health topics, and further align experiential learning with formal curricula addressing ethics, health equity, and systems-based practice. To translate these gaps into actionable student work for future cohorts, several innovation challenges are currently under consideration for student projects aimed at improving the delivery of correctional healthcare. A single device capable of managing multiple continuous glucose monitors is one such challenge in need of development made particularly relevant by the restriction on smartphone access for individuals who are incarcerated.

At the individual level, this experience may influence how trainees conceptualize healthcare delivery, professional responsibility, and health equity. While only a subset of learners is likely to pursue careers in correctional medicine, participants may gain a more informed, comprehensive understanding of the role correctional facilities play within the broader healthcare system, including recognition of incarceration as a social determinant of health.^5^ This experience allows trainees to observe first-hand how institutional structures shape care rather than encountering health equity as a theoretical concept. Although limited to a single program, structured correctional facility visits appear to be a feasible addition to pre-health training and warrant consideration by educators designing exposure to structural determinants of health at the pre-health and medical education levels.

### Acknowledgments

The authors thank the partnering physicians and leaders who contributed their time, mentorship, and expertise to the educational experiences described in this article, including Dr. Anthony Chang, Dr. Adam Kalawi, Debra Beauregard, and Tiffani Ghere. We also acknowledge the Orange County correctional facility clinical team and the correctional officers for their collaboration and support.

### Conflict of Interest

The authors declare that they have no conflicts of interest.
